# The Role of Non-Gaussian Models of Diffusion Weighted MRI in Hepatocellular Carcinoma: A Systematic Review

**DOI:** 10.3390/jcm10122641

**Published:** 2021-06-15

**Authors:** Liberatore Tramontano, Carlo Cavaliere, Marco Salvatore, Valentina Brancato

**Affiliations:** IRCCS SDN, 80143 Naples, Italy; liberatore.tramontano@synlab.it (L.T.); direzionescientifica.irccssdn@synlab.it (M.S.); valentina.brancato@synlab.it (V.B.)

**Keywords:** hepatocellular carcinoma, magnetic resonance imaging, diffusion weighted MRI, non-Gaussian DWI, Intravoxel Incoherent Motion, Diffusion Kurtosis Imaging, Stretched Exponential

## Abstract

The importance of Diffusion Weighted Imaging (DWI) in hepatocellular carcinoma (HCC) has been widely handled in the literature. Due to the mono-exponential model limitations, several studies recently investigated the role of non-Gaussian DWI models in HCC. However, their results are variable and inconsistent. Therefore, the aim of this systematic review is to summarize current knowledge on non-Gaussian DWI techniques in HCC. A systematic search of the literature, including PubMed, Google Scholar, MEDLINE, and ScienceDirect databases, was performed to identify original articles since 2010 that evaluated the role of non-Gaussian DWI models for HCC diagnosis, grading, response to treatment, and prognosis. Studies were grouped and summarized according to the non-Gaussian DWI models investigated. We focused on the most used non-Gaussian DWI models (Intravoxel Incoherent Motion (IVIM), Diffusion Kurtosis Imaging (DKI), and Stretched Exponential—SE). The quality of included studies was evaluated by using QUADAS-2 and QUIPS tools. Forty-three articles were included, with IVIM and DKI being the most investigated models. Although the role of non-Gaussian DWI models in clinical settings has not fully been established, our findings showed that their parameters may potentially play a role in HCC. Further studies are required to identify a standardized DWI acquisition protocol for HCC diagnosis, grading, response to treatment, and prognosis.

## 1. Introduction

Hepatocellular carcinoma (HCC) is the most common form of primary liver cancer in the world and is one of the leading causes of cancer-related mortality worldwide [1]. HCC development is characterized by extremely heterogeneous pathogenic mechanisms, epidemiology, and underlying diseases from each etiology. This makes HCC diagnosis difficult at an early stage, thus affecting the choice of an effective therapeutic approach [2,3,4]. Imaging plays a key role in HCC and all major clinical practice guidelines recommend the use of Computed Tomography (CT) and Magnetic Resonance Imaging (MRI) as the first-line modalities for diagnosis and staging of HCC [5]. Multiparametric MRI is an excellent non-invasive tool for HCC diagnosis, grading, response to treatment, and prognosis. This because it combines morphological MRI sequences (such as T1 and T2 weighted) with functional methods such as diffusion weighted imaging (DWI) and dynamic contrast-enhanced imaging, with the latter involving the use of hepatobiliary contrast agents [5,6,7,8]. Among them, DWI is a promising tool in HCC assessment and has the benefit of not requiring contrast injection since it relies on the diffusion phenomenon associated with the microscopic motility of water molecules in tissues [9]. Depending on how the motility of water molecules is limited by the tissue structure, the DWI signal intensity varies, and this may give information that is functional to HCC diagnosis, grading, response to treatment, and prognosis [10].

Several diffusion MRI models have been explored for HCC diagnosis, grading, response to treatment, and prognosis. The most commonly used one is the conventional mono-exponential DWI model, which presumes that the probability function of the water molecules displacement follows a Gaussian distribution. This model provides a single parameter, called Apparent Diffusion Coefficient (ADC), which represents an average diffusion value [11].

Although several studies used the mono-exponential DWI model in HCC [12,13,14,15,16,17], this model is based on assumptions that are often inaccurate since in vivo water diffusion is more complex, may be anisotropic, and often presents non-Gaussian behavior. Given the above, ADC value may not be associated with the true tissue characteristics. Diffusion Tensor Imaging (DTI) is an MRI technique based on Gaussian diffusion model and accounting for the diffusion anisotropy by means of additional gradients. Through providing additional information on anisotropy diffusion and total diffusion orientations, DTI can achieve a more precise ADC calculation thanks to scalar parameters, such as Fractional Anisotropy (FA), Mean Diffusivity (MD), Radial Diffusivity (RD), and Axial Diffusivity (AD) [18,19]. However, only a few studies investigated the application of liver DTI, and it remains unknown if diffusion in HCC is isotropic or anisotropic [20]. Concerning the non-Gaussian behavior of in vivo water diffusion, when many b-values are used to measure diffusion signal, considerable displacements from the mono-exponential model are detected. In particular, at low b-values (≤200 s/mm^2^), the signal attenuation is greater than expected (and, consequently, the calculated ADC is higher), while at larger b-values (≥1500 s/mm^2^), signal attenuation is often lower than expected (and, consequently, the calculated ADC is lower). To better describe this trend, several non-Gaussian diffusion models have been proposed and explored, with their associated parameters aiming at better profiling physiologic and pathologic properties of the in vivo tissue, such as cellularity, vascularity, and heterogeneity [21,22,23]. The most investigated non-Gaussian DWI models in HCC applications are the Intravoxel Incoherent Motion (IVIM) [24,25], the Diffusion Kurtosis Imaging (DKI) [26], and the Stretched Exponential (SE) [27]. The first is a bi-exponential model which can simultaneously quantify the diffusion of water molecules and the microcirculation perfusion in living tissues, thus compensating for the inability of the mono-exponential model to differentiate between the diffusion of water molecules and the blood perfusion. IVIM-related parameters are the pure diffusion coefficient (D), which reflects the diffusion of pure water molecules, the pseudo-diffusion coefficient (D*) reflecting the diffusion movement of capillary microcirculation perfusion, and the perfusion fraction (f), which represents the volume ratio between the perfusion effect of local microcirculation and diffusion effect in overall [24,25]. The DKI model quantifies the deviation of tissue diffusion from a Gaussian behavior due to diffusion barriers constituted by cell membranes and organelles or other hindrance due to complex and restricted structures in tissues. This model evaluates the microstructural complexity of tissues better than standard DWI, and its associated parameter is DK, which is an analog of ADC corrected for non-Gaussian behavior and K, the kurtosis coefficient expressing the displacement from gaussianity [26]. Lastly, the SE model considers the deviation from mono-exponential trend by using a Stretched Exponential equation described by two parameters: α is the so-called heterogeneity index and describes the deviation from a single exponential decay, while DDC is the distributed diffusion coefficient, which can be considered as a weighted sum over a distribution of ADCs that comprises the multi-exponential decay properties [27]. Characteristics of non-Gaussian models are summarized in Supplementary Materials Table S1.

Although several studies on HCC aimed at investigating the role of non-Gaussian DWI models in HCC, their results suffer from inconsistency, insignificance and the lack of a clear physical interpretation of non-Gaussian parameters [28,29,30,31,32]. For this reason, the aim of this systematic review was to summarize the existing knowledge on the use of non-Gaussian DWI models in HCC.

## 2. Materials and Methods

### 2.1. Search Strategy and Selection Criteria

A systematic search of the literature was performed to identify original articles that evaluated the role of any diffusion metrics arising from any non-Gaussian DWI models for HCC diagnosis, grading, response to treatment, and prognosis. The most relevant scientific electronic databases (PubMed, Google Scholar, MEDLINE, and ScienceDirect) were comprehensively explored and used to build the search. Only studies published since 2010 were selected (to November 2020). The search strategy included the key terms listed in Supplementary Materials Section S2. The literature search was restricted to English language publications and studies involving human participants.

Two reviewers, after having independently screened the identified titles and abstracts, assessed the full text of articles that evaluated the use of at least one non-Gaussian DWI model between IVIM, DKI, and SE for HCC diagnosis, grading, response to treatment, and prognosis, and that were not review articles.

For articles meeting these criteria with full text available, the following further selection criteria had to be fulfilled: involvement of adult patients (age > 18); involvement of patients with HCC confirmed by pathology and/or surgery and/or overall analysis combined with medical history, clinical symptoms, and various imaging data; presence of information about DW-MRI protocol. Moreover, studies were excluded if they performed analyses on mixed patients (e.g., groups of patients with multiple hepatic malignant diseases), not allowing to draw conclusions only about HCC patients. However, studies belonging to this category were maintained if values of diffusion metrics were reported.

### 2.2. Planning and Conducting the Review

After the selection procedure, selected articles were analyzed by two reviewers, and data useful for conducting the systematic review were collected in a predesigned sheet. Extracted data will include the following: study characteristics (first author name, publication year, study design, in particular prospective or retrospective, and number of included patients), number of HCC lesions, clinical purpose, diffusion acquisition details, diffusion MRI model/s evaluated, diffusion MRI metric/s evaluated, information on the ROI placement, and main findings.

Studies were classified and analyzed according to the non-Gaussian model investigated. If more than one non-Gaussian diffusion model was investigated in the same study, each model was treated as belonging to a separated study. Moreover, if a study had multiple purposes, each aim was discussed separately in the results section.

This systematic review was conducted in accordance with the Preferred Reporting Items for Systematic Reviews and Meta-Analyses (PRISMA) statement (see Supplementary Materials for PRISMA Checklist) [33].

### 2.3. Quality Assessment

The quality of the included studies was evaluated by using the QUADAS-2 tool for the diagnostic studies and the QUIPS tool for the prognostic studies. The quality of each study was evaluated by two reviewers independently and any disagreement was resolved by consensus. Concerning QUADAS-2 tool, four domains were scored: (1) patient selection, (2) index test, (3) reference standard, and (4) flow and timing. Items were scored as “yes”, “no”, or “unclear” [34]. Concerning QUIPS, six domains were scored: (1) selection of study participants, (2) study attrition, (3) prognostic factor measurement, (4) outcome measurement, (5) study confounding, and (6) statistical analysis and reporting. For each domain, the responses “yes”, “partial”, “no”, or “unsure” for three up to seven items within each domain were combined to assess the risk of bias. An overall rating for each domain is assigned as “high”, “moderate”, or “low” risk of bias [35,36].

## 3. Results

### 3.1. Study Selection

**Figure 1:**
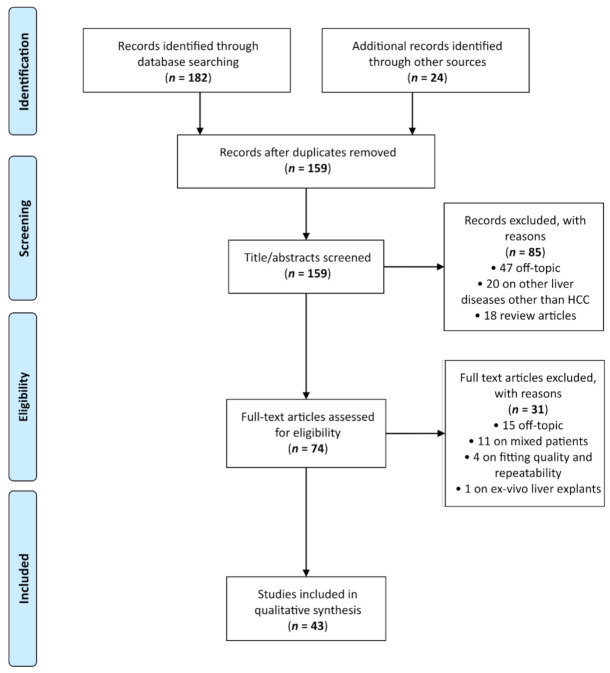
Preferred Reporting Items for Systematic Reviews and Meta-Analyses (PRISMA) flow diagram.

A total of 182 articles were retrieved by scientific electronic databases search. Twenty-four additional articles were found through article references, bringing the total number of records suitable for further evaluation to 206. After the exclusion of duplicates there were 159 articles left for investigation. By scanning the title and abstract of these records, 85 records were excluded because they clearly did not match the inclusion criteria (47 were not in the field of interest, 18 were review articles, 20 involved patients with other liver diseases other than HCC). Seventy-four articles were evaluated on their full text. Of these articles, 31 records were excluded based on the inclusion criteria (15 were off-topic, 11 were excluded since they included HCC patients but did not perform analyses only on HCC patients, four were on fitting quality and repeatability of non-Gaussian parameters, and one was not in vivo but on ex vivo liver explants). Finally, 43 records were included for qualitative synthesis. The PRISMA flow diagram of included studies according to the inclusion and exclusion criteria is reported in Figure 1.

### 3.2. Characteristics of Included Studies

Characteristics of the 43 selected articles selected are reported in Table 1. All selected studies were targeted to adults and the median number of individuals (±absolute deviation) was 56 ± 32.7, while the median number of HCC lesions (±absolute deviation) was 54 ± 29.8. Study designs were 55.8% (24/43) prospective and 44.2% (19/43) retrospective. Thirty-two studies involved the IVIM model (74.4%), nine involved the DKI model (20.9%), one involved the SE model (2.35%), and the remaining one involved both the SE and IVIM model (2.35%). Due to the larger number of IVIM studies with respect to those on DKI and SE, Section 3.3, “Studies on IVIM”, is further divided into subparagraphs to facilitate reading.

### 3.3. Studies on IVIM

Thirty-three of the included studies investigated the role of IVIM model in HCC, of which one investigated also SE model [31]. Among the 33 studies, 11 investigated IVIM for HCC diagnosis, namely for HCC detection respect to normal liver parenchyma or other types of hepatic lesions (either benign or malignant), and seven evaluated the power of IVIM parameters for HCC histological grading. Among the 15 remaining studies, six assessed the usefulness of IVIM for the response of HCC to therapy [40,42,48,66,69,73], five evaluated if IVIM could be associated with prognostic factors [51,52,56,58,67], and four explored IVIM model for multiple aims [37,41,53,64] (two investigated response to therapy and survival [53,64], one on grading and prognostic factors [41], and the remaining one on diagnosis and grading [37]).

#### 3.3.1. Diagnosis

Choi et al. [61] performed a study involving patients with HCC, intrahepatic cholangiocarcinoma (IHCC), and hemangiomas, combined HCC–IHCC and metastases, and found that all IVIM parameters except D* were able to characterize HCC from hemangioma, IHCC, and metastasis. Similar results on D and D*, and controversial results on f, were obtained in the study by Qu et al. [71] on patients with HCC, IHCC and metastases. Different from Choi et al., any of the IVIM-associated parameters were able to reflect differences between HCCs and metastases. Two studies [37,49] focused on differentiating between HCC and IHCC and found similar results on D with respect to studies comparing HCC with other multiple lesions mentioned above [61,71], while results on D* and f were controversial. Wei et al. [57] aimed at differentiating HCC from IHCC in the setting of liver cirrhosis, finding results for D that were in agreement with those of the previous studies [37,49,61,71], with D showing higher diagnostic performances than ADC. However, any significant results were found concerning D* and f. In a study comparing HCC with hemangioma and metastasis [72], D and f were able to detect differences between HCC and hemangioma group, while no IVIM metrics were useful for differentiating between HCC and metastases. Kim et al. performed a study on a similar patient cohort and confirmed the utility of D and f in distinguishing between HCC and hemangioma [31]. Interestingly, f was able to differentiate HCCs from metastases and showed the largest AUC with respect to the other diffusion parameters. Findings in line with those by Zhu et al. [72] were obtained by Watanabe et al. [74] in a study on a similar patient cohort. Notably, they also included liver cysts among the benign lesions. Two studies evaluated the diagnostic efficacy of IVIM parameters in differentiating HCC from focal nodular hyperplasia (FNH) [62,68]. Both found that D could be used for discriminating between HCC and FNH, while f was not a useful marker for that purpose. Conflicting results were found concerning D*. However, both studies agreed on the comparable diagnostic utility of ADC and D, revealing no added value of IVIM parameters for HCC diagnosis. Two studies by Hectors et al. [55,65] investigated differences in IVIM parameters in both HCC and liver parenchyma of the same patients. The first study revealed that all three IVIM parameters were able to detect HCC, also suggesting that IVIM improved HCC characterization compared to ADC. Completely opposite findings were obtained in the second study. Peng et al. and Zhu et al. [37,72] also evaluated IVIM parameters for differentiating normal liver and HCC, but they investigated normal liver parenchyma from healthy volunteers. Both found that IVIM parameters of the HCC tissues were all able to distinguish HCC from the normal liver tissues, even if controversial results were found concerning D*.

#### 3.3.2. Grading

Shan et al. [38] aimed at evaluating the diagnostic value of IVIM in discriminating histologic grades of HCC with respect to conventional ADC. They found that D and f were able to discriminate from well-, moderately, and poorly differentiated HCCs, and showed a descending trend with the increase of the HCC grade. However, they were not superior to ADC concerning diagnostic performances for discriminating HCC histologic grades. Any results were obtained concerning D*. Similar results were obtained by the same research group in a previous study [63], and also by Wu et al. [39]. Decreasing values of D with grade increasing were also found in studies by Woo et al. [75], Peng et al. [37], and Granata et al. [28], even if findings on f were controversial. Interestingly, Woo et al. [75] found that D values from IVIM was better than ADC for HCC grading. Different from the previous studies, Sokmen et al. [50] investigated the diagnostic accuracy of IVIM parameters for HCC grading by grouping patients in low- and high-grade groups. They found that both D and f reflected HCC grade, with D positively and f inversely correlated with tumor grade. Zhu et al. [59] confirmed results on D, which, notably, was better than ADC in differentiating the low-grade from high-grade HCC with a good correlation between the ADC and D values and the histological grades. Although D* and f were not able to distinguish between different HCC grades, a significant negative correlation was found between D* and HCC grade. However, differently from Sokmen et al., they included patients with G2 (according to Edmondson–Steiner grade) in the high-grade group. Shi et al. [41] aimed to predict histologic grade by using histogram-based IVIM parameters. They found that many of them were able to distinguish between low- and high-grade HCC patients and correlated with the histopathologic grade. However, only two of them were associated with D*.

#### 3.3.3. Response to Therapy

Among studies assessing the usefulness of IVIM the response of HCC to therapy, Jia et al. [40] found that D could be helpful in predicting HCC response to transarterial chemoembolization (TACE) and was a significant predictor of response to therapy both univariately and in multivariate analysis, including also parameters derived from amide proton transfer. Park et al. [73] also assessed the usefulness of IVIM in the response of TACE in HCC and found that D* was able to distinguish patients with good lipiodol uptake from those with a poor one. Server et al. [48] aimed to evaluate IVIM power for HCC response to transarterial radioembolization (TARE) or TACE and found that D values and f-values were able to reflect treatment response of HCC patients. Any results were found on D*. Similar results were obtained by Murtz et al. [66] to evaluate HCC response after locoregional therapy. In particular, D values increased after therapy in the responsive group, whereas f-values decreased. They also found that the differentiation between responders and non-responders was better assessed by using D than the conventional ADC. Differently from Server et al. [48], they involved HCC patients treated also with transarterial ethanol–lipiodol embolization therapy. Moreover, they used a simplified version of the IVIM model. Shirota et al. [69] aimed at evaluating the association between the therapeutic outcomes of sorafenib for HCC and IVIM metrics. They found that the D value before treatment could be a valuable biomarker for predicting the therapeutic effects of sorafenib for HCC. Two studies aimed to assess response to therapy of HCC patients performing a histogram analysis of IVIM metrics [42,53]. Hectors et al. involved HCC patients treated by yttrium 90 radioembolization and found that D* and D could be used to evaluate HCC response to radioembolization. In study by Wu et al. performed on patients treated with TACE, several histogram-based IVIM parameters were found to be useful to differentiate responders from non-responders. In a previous study with a similar setting, Wu et al. [64] found that D ratio 24–48 h after TACE was an independent predictor for response to TACE for HCC.

#### 3.3.4. Prognosis

Other authors investigated if IVIM parameters could be associated with prognostic factors. Three studies evaluated if IVIM could predict microvascular invasion (MVI) in HCC patients. Two studies showed comparable results and found that D was a preoperative predictor of MVI in HCC patients and was superior to ADC [51,58]. Any significant results were found on D* and f. Results emerging by a histogram analysis performed by Li et al. [56] were also in line with those of the previous two studies and revealed that D 5th percentile had significantly higher accuracy than the ADC for differentiation of HCC patients with and without MVI.

Shi et al. [41] found that several histogram-based IVIM parameters (D mean, f 70th percentile, D 40th percentile, and D* 75th percentile) were able to predict the expression of Ki67 and capsule formation of HCC. Zhang et al. [52] aimed to evaluate IVIM parameters to predict tumor recurrence after hepatectomy in patients with HBV related HCC and found that D was a valuable biomarker for the preoperative prediction of recurrence after hepatectomy in HCC patients. D was also associated with progression free survival of HCC TACE-treated patients in study by Wu et al. [64], even if similar performances were shown by ADC. In a subsequent study performed by the same group, they investigated if histogram-based IVIM features could be associated with HCC time to progression, but any significant results were found [53]. Finally, Kakite et al. [67] assessed the diagnostic performance of IVIM parameters for prediction of complete tumor necrosis, finding a significant positive correlation between D and tumor necrosis. An opposite trend was found for f. D had the highest AUC for predicting complete tumor necrosis but also f- and ADC values were significantly higher when comparing all tumors to liver parenchyma.

### 3.4. Studies on DKI

Nine of the included studies investigated the role of DKI model in HCC. Specifically, two of them investigated DKI for HCC diagnosis [45,54] and two for HCC grading [43,44]. Among the remaining five, two investigated the role of DKI for prediction of response to treatment [47,70], two assessed if DKI could be associated with prognostic factors [46,60], and the remaining one [30] investigated the power of DKI for both MVI prediction and histological grading assessment. Wang et al. [43] investigated if DKI parameters could be used for HCC pathological grading and found that both K and DK were able to characterize HCC with low differentiation from HCC with medium-to-high differentiation. K and DK were positively and negatively correlated with the pathological grade, respectively. Moreover, Wu et al. [44] found similar results, with higher-grade HCC showing lower DK and higher K values than lower-grade HCC. These findings are consistent both with results from Wang et al. [43] and those by Cao et al. [30]. Authors agreed that decreased DK and increased K values in higher grade HCC could be associated to the increased cellular density and architectural complexity of these lesions with respect to the lower-grade HCC lesions. Concerning studies on HCC diagnosis, Budjan et al. [54] explored DKI parameters for differentiating between benign and malignant lesions, finding that DK was able to distinguish HCC from benign lesions. Moreover, Jia et al. [45] used DKI parameters for differentiating between HCC and benign nodules (FNH, hemangioma, and HCA), highlighting that both K and DK were able to differentiate among benign lesions and HCC. According to the authors, these results could be associated to the ability of DKI of providing information about heterogeneity and irregularity of tissue components, which are more pronounced in malignant lesions. However, in both studies, it was not possible to affirm that DKI parameters were better than those of the ADC of conventional DWI in differentiating HCC from benign hepatic nodules. Among studies investigating the role of DKI in identifying HCC prognostic factors, two studies [30,60] found that K was a promising tool for MVI prediction and outperformed conventional ADC values for predicting MVI and for the assessment of early tumor recurrence risk. According to these studies, this could be attributed to the more complex and heterogeneous microenvironment introduced by MVI. Yuan et al. [46] evaluated the ability of DKI to predict the recurrence of early stage single nodules of HCC treated by radiofrequency ablation (RFA). This study revealed that the prediction efficacy of DKI was better than that of DWI, with K being the most sensitive predictor among DKI parameters. Among studies evaluating DKI for prediction of response to treatment, Luo et al. [47] believed that both DKI and ADC were complementary in the post TACE evaluation; in fact, DKI allowed the evaluation of the presence of tumor necrosis or recurrence but not of tissue perfusion changes, thanks to ADC variations with different b-values. Interestingly, they evaluated also other metrics than K and DK, and found that radial diffusivity, axial diffusivity, and DK of tumor tissues in the patients after TACE were significantly increased. The opposite happened for axial kurtosis, kurtosis fractional anisotropy of kurtosis. They found any significant results for K. Goshima et al. [70] found that K was able to assess the hypervascular HCC response to treatment (RFA and TACE), outperforming conventional ADC. In particular, they compared K and ADC values between viable (untreated or locally recurrent HCCs) and non-viable (completely necrotic HCCs) groups and concluded that DKI can be a new option for the evaluation of response to treatment of HCC.

### 3.5. Studies on SE

Two of the included studies aimed at investigating if SE parameters could be useful to characterize HCC from other liver lesions [29,31]. Kim et al. [31] found that DDC was able to distinguish HCC from hemangioma, but not from metastases. Findings by Noda et al. [29] were in line with these results. In particular, they also found that DDC differed between HCC and benign lesions, including hemangioma, and were similar between HCC and metastases. According to the authors, results on DDC may be associated to the higher density of cells and stroma of malignant lesions that restricts the movement of water in tissue with respect to benign lesions. Notably, both studies agreed on the inability of α to differentiate benign and malignant hepatic lesions. Since this parameter is associated with intravoxel water molecular diffusion heterogeneity, authors suggested that it could be due to the high number of cell components that characterize both benign and malignant hepatic lesions [29,31].

### 3.6. Quality Assessment

**Figure 2:**
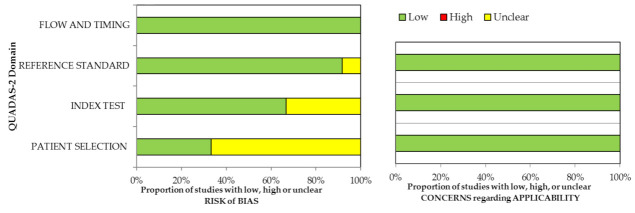
Quality assessment using QUADAS-2 tool for diagnostic studies.

**Figure 3:**
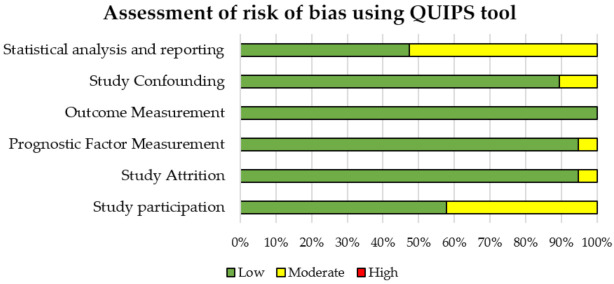
Quality assessment using QUIPS tool for prognostic studies.

The overall quality of included studies was considered good for our purposes, both considering QUADAS-2 and QUIPS results. Results of quality assessment are shown in Figure 2 and Figure 3 and reported in Supplementary Materials Tables S2 and S3. Concerning the QUADAS-2 assessment, the risk of bias was ranked low or moderate across all the diagnostic studies, for all the four QUADAS-2 domains. The applicability concerns were ranked low across all the diagnostic studies. Similarly, concerning the QUIPS assessment, the risk of bias was ranked low or moderate across all the prognostic studies, for all the six QUIPS domains.

## 4. Discussion

In this systematic review we aimed at investigating the role of the non-Gaussian DWI models in HCC. In the last decade, DWI forcefully entered the clinical routine of HCC diagnostic, due to the ability of the diffusion MRI techniques of providing a quantitative assessment of HCC lesions, without the use of any contrast agent [7,8,10]. However, due to the inability of the conventional DWI model to depict the heterogeneous behavior of water diffusion in tissues, several non-Gaussian models were investigated attempting to correctly depict the underlying water diffusion signal and to consequently explore if their associated parameters could provide additional information on HCC with respect to conventional DWI model [22,31,32,46,51,68]. However, results concerning the benefits of using non-Gaussian models for HCC assessment are often controversial or unsatisfactory. In this scenario, we performed this systematic review to provide new insights and help to reach a common view on the use of the most common non-Gaussian DWI models (IVIM, DKI, and SE) for HCC diagnosis, grading, response to treatment, and prognosis. Forty-three studies from 2010 onwards were examined. Concerning IVIM-related parameters, D has been shown to have a great potential for differentiating HCC from other hepatic lesions, either malignant (e.g., IHCC) or benign (e.g., hemangioma and FNH), as well as from normal liver parenchyma [37,49,61,62]. Interestingly, this IVIM parameter has shown better diagnostic performances than ADC in several included studies [49,57,61,68,71]. Conversely, results concerning the perfusion-related parameters D* and f were inconsistent or controversial. These fluctuations of D* and f-values could be related to the location of the lesions and their different blood supply [59,72,76,77], an issue that should be taken into account before performing analyses [72]. Notably, the differentiation of HCC and metastasis revealed unsatisfactory results also for D, and this could be attributed to the origin of metastases from primary neoplasms (e.g., gastrointestinal, lung, breast, and genitourinary) that may give rise to variability at the cell-density and microcirculation level [78]. D was also able to characterize HCC grade, while results concerning f and D* were controversial and often not consistent for HCC grading [49,61]. Again, this could be attributed to the different blood supply across HCC grades, which has been shown to be highly variable, especially in HCC with medium or high differentiation [79,80]. However, these results should be carefully interpreted since the study setting was different across studies on HCC grading. In particular, grading classification systems varied across studies, with some of them using the Edmondson–Steiner grading system [81] and others the WHO classification grading system (well-, moderately, or poorly differentiated HCC) [82]. Moreover, among the studies investigating the differences between low- and high-grade HCC, some included borderline HCC lesions in the low-grade group and others in the high-grade group [83]. D was also found to be an important biomarker for prediction and assessment of response to treatment and a predictor of MVI and other prognostic factors. To summarize, results on IVIM studies produced variable findings, mainly relating to perfusion-associated parameters. There may be several reasons for the discrepancies in these results: the previously mentioned different blood supply across lesions, the different choice of data-fitting algorithms, the missing reached consensus on the number and range of b-values used for IVIM, the instability and poor reproducibility of D* and f, and also the ROI placement [84,85]. To our knowledge, only few studies approached these issues. Kakite et al. [84] found poor reproducibility of D* and f in HCC and liver parenchyma. According to Koh et al. [10], six to eight b-values in total, with four or more within the perfusion range should be used to better quantify IVIM perfusion parameters. Wei et al. found that different ROI positioning methods significantly affect the IVIM parameters concerning diagnostic performances in grading HCC [85]. Given the above, measurement reproducibility and accuracy of IVIM-derived parameters should be improved. Although few studies on DKI were detected, promising results were found concerning both K and DK metrics, for both the diagnosis and prognosis of HCC [45,46,54,70]. However, the low number of included studies prevented us from assessing the effective utility of DKI in HCC. Moreover, it should be noted that two of these used b-values up to 1000 mm^2^/s; however, the usage of b-values at least equal to 1500 is suggested to better appreciate the non-Gaussian behavior [26,86]. Although the promising results concerning DDC from SE for HCC characterization with respect to other hepatic lesions, this was not sufficient to establish a clear role of this metric for HCC assessment due to the survival of only two studies on the SE model after the selection process. However, other studies showed SE power for diagnosis and characterization of several liver diseases [87,88,89].

To our knowledge, this is the first systematic review aiming at summarizing the role of non-Gaussian diffusion MRI models in HCC. To date, systematic studies were performed on conventional diffusion metrics for HCC detection and prognosis [15,90,91]. Surov et al. performed a meta-analysis to assess the role of DWI in prediction of tumor grading and MVI in HCC, also including D in the investigated metrics [15]. Wu et al. performed a meta-analysis evaluating the IVIM model in differentiating focal liver lesions, also including HCC [78]. Tao et al. performed a review on the role of IVIM in liver diseases, summarizing also its role in HCC [92]. Notably, Granata et al. performed a review specific on HCC, including both IVIM and DKI, but the study was non-systematic and the role of SE model was not discussed [32].

Summarizing, the main findings and conclusions of the selected studies varied from each other, often showing inconsistencies and not a clear idea about the actual usefulness and the effective power of the non-Gaussian diffusion MR biomarkers, especially IVIM perfusion parameters. Despite the above, our study revealed that the most powerful non-Gaussian diffusion metrics were D from IVIM, DK and K from DKI, and DDC from SE. It could be interesting to evaluate if non-Gaussian parameters combined with other clinical or imaging parameters could improve the diagnosis and/or prognosis of HCC, as has been previously demonstrated for the added value of age and serum alpha-fetoprotein levels [38], and also similarly to what was found by Wu et al., or APT imaging [39]. Characteristics of the included studies, such as patient treatment, study setting of studies with similar purposes, diffusion sequence parameters, diffusion metrics for the same diffusion model, fitting models, analysis methods, and ROI placement methods, were highly variable across studies, preventing us from performing a meta-analysis to quantitatively confirm results obtained from the qualitative synthesis. This is directly linked to the lack of studies concerning reproducibility of non-Gaussian models for HCC assessment [84,93]. However, selected studies were affected by other limitations, such as the small and often unbalanced patient sample. Another interesting point to be raised is that it should be noted that a large part of the included studies was retrospective, and they are supposed to be more bias-affected. Thus, in future studies involving non-Gaussian parameters for in HCC, the reproducibility of the parameters should be investigated. Moreover, further prospective studies involving larger populations are required to validate findings from studies included in this review.

Finally, it is worth noting that the application of the recently introduced radiomics approach to non-Gaussian DWI models could be a useful tool for HCC diagnosis, grading, response to treatment, and prognosis [94,95]. In this context, four of the selected studies [41,42,53,56] investigated first-order features arising from histograms, but any of the selected studies investigated more complex radiomic features.

## 5. Conclusions

Although the role of non-Gaussian DWI metrics in HCC remains a debatable issue, this systematic review summarized the current literature on non-Gaussian DWI models for HCC diagnosis, grading, response to treatment, and prognosis and highlighted the pros and cons, with the latter mainly related to the lack of standardization in the diffusion protocol. It may serve as a starting point for future studies evaluating non-Gaussian DWI metrics performances to support a more precise biophysical interpretation of their parameters with the objective of identifying a standardized HCC DWI protocol for clinical purposes.

## Figures and Tables

**Table 1:** Characteristics of included studies. Abbreviations: NS = number of subjects; HCC = hepatocellular carcinoma; IHCC = intrahepatic cholangiocarcinoma; HV = healthy volunteers; HCA = hepatocellular adenoma; TR = repetition time; TE = echo time; SG = interslice gap; vs. = voxel size; ST = slice thickness; ROI = Region of Interest; P = prospective; R = retrospective; IVIM = Intravoxel Incoherent Motion; DKI = Diffusion Kurtosis Imaging; SE = Stretched Exponential; ADC = Apparent Diffusion Coefficient; D = IVIM True Diffusion; D* = IVIM pseudo-diffusion coefficient; f = IVIM perfusion fraction; DK = DKI diffusion coefficient corrected for kurtosis; K = DKI kurtosis; DDC = SE distributed diffusion coefficient; α = SE heterogeneity index; SD = study design; NR = not reported.

Reference	Year	NS	HCC Lesions	SD (P/R)	Clinical Purpose	Diffusion Acquisition Details	Non-Gaussian Diffusion Models	b-Values (s/mm^2^)	Non-Gaussian Parameters	ROI Info	Main Findings
Noda et al. [29]	2020	56 HCC	19	P	Diagnosis	TR = 5000 ms, TE = 57 ms; FOV = 40 × 32 cm; matrix = 96 × 96; EPI factor, 2.0; ST = 6 mm, SG = 1 mm; slices = 30, VS = NR	SE	0, 10, 25, 50, 75, 100, 200, 500, and 800	DDC, α	ROIs on the entire hepatic lesions in DWI images; manual segmentation.	DDC values were higher in HCC than in benign lesions and similar to those in metastases
Peng et al. [37]	2020	82 (55 HCC, 10 IHCC, and 17 HV)	55	P	Diagnosis and grading	TR = 3529 ms; TE = 60.8 ms, FOV = 36 × 36–40 cm × 40 cm, matrix of 128 × 160, ST = 5 mm, SG = 0,5 mm, vs. = NR	IVIM	0, 20, 40, 80, 100, 200, 400, 800, and 1000	D, D*, f	ROIs on the largest solid areas of the lesions avoiding regions of necrosis and hemorrhage; NR.	D lower in HCC than IHCC and inversely correlated with grade; D* higher in HCC than IHCC and not correlated with HCC grade; f not useful for differentiation HCC/IHCC but positively correlated with HCC grade
Shan et al. [38]	2020	117 HCC	120	R	Grading	TR = 6000–10,000 ms; TE = 56 ms; FOV = 30 × 30 cm, FA = 90°; matrix size = 128 × 128; BW = 250 kHz/pixel, ST = 5 mm, SG = 1 mm, VS = NR	IVIM	11 b-values (b = 0, 30, 50, 100, 150, 200, 300, 500, 800, 1000, and 1500) and 2 b-values (b = 0, 800)	D, D*, f	ROIs on the axial b800 images of solid components; manual segmentation.	D and f inversely correlated with HCC grading and significantly different among HCC grades. D* not significant.
Wu et al. [39]	2020	88 HCC	88	P	Grading	TR = 2500 ms, TE = 58.8 ms; FOV = 380 × 380 mm, FA = 90; matrix = 128 × 128; number of excitations = 2–6; ST = 5 mm; SG = 1.0 mm; slices = 20, vs. = NR	IVIM	0, 20, 40, 80, 160, 200, 400, 600, 800, and 1000	D, D*, f	ROIs on the solid part of the tumor avoiding cystic degeneration, necrosis, and bleeding; manual segmentation.	D and f inversely correlated with HCC grading and significantly different among HCC grades. D* not significant.
Jia et al. [40]	2020	56 HCC	56	P	Response to treatment	TR = 2500 ms; TE = 5 8.8 ms; FOV = 380 × 380 mm, FA = 90; matrix = 128 × 128; number of excitations = 2–6; ST = 5.0 mm; SG = 1 mm; slices 20, VS = NR	IVIM	0,20, 40, 80, 160, 200, 400, 600, 800, and 1000	D, D*, f	ROIs on the solid part of the tumor avoiding cystic degeneration, necrosis, and bleeding; manual segmentation.	D may be useful for predicting the response of intermediate-stage HCC to TACE.
Shi et al. [41]	2020	52 HCC	52	P	Grading and prediction of prognostic factors	TR: 4294.0 ms, TE:67.1 ms, FA = 90°, ST = 5 mm, SG = 1.0 mm, matrix = 200 × 256,VS = NR	IVIM	0, 10, 20, 30, 40, 60, 80, 100, 200, 500, and 800	D, D*, f (histogram analysis)	ROIs on the whole lesion of HCC in D map with T2WI image as a reference; semi-automatic segmentation.	Histopathologic grade, Ki67 expression status, and capsule formation can be predicted by IVIM histogram metrics.
Hectors et al. [42]	2020	24 HCC	25	P	Response to treatment	TR = one respiratory cycle, TE = 74–81 ms, FOV: 340–450 × 220–305 mm, matrix 160 × 80–132, GRAPPA 2, ST = 7 or 8 mm; at 3.0T was performed during free breathing, TR = 4500 ms, vs. = NR	IVIM	0, 15, 30, 45, 60, 75, 90, 105, 120, 135, 150, 175, 200, 400, 600, 800	D, D*, f (histogram analysis)	ROIs on slices on which the lesion was visible, avoiding partial volume effects. Liver ROI placed in a single slice mid liver away from the tumor and large vessels; manual segmentation.	IVIM histogram features together with perfusion features can be used to predict HCC response to radioembolization.
Wang et al. [43]	2020	128 HCC	128	P	Grading	TR = 3300 ms, TE = 88 ms, FOV = 380 × 420 mm, matrix size = 168 × 105, FA = 90°, ST = 5 mm; SG = 1.5 mm, VS = NR	DKI	0, 800, and 1500	DK, K	ROIs on solid parts of the largest lesions avoiding large vessels, bile ducts, necrosis and artifacts; manual segmentation.	High-grade HCCs have higher K values and lower DK values than low-grade HCCs
Wu et al. [44]	2020	88 HCC	88	P	Grading	TR = 2500 ms; TE = 58.9 ms; FOV = 360 × 280 mm; matrix = 128 × 128, FA = 90; excitations = 2; ST = 5.0 mm; SG = 0.5 mm; slices = 24, vs. = NR	DKI	0, 1000, and 2000	DK, K	ROIs on the solid part of the tumor; manual segmentation.	High-grade HCCs have higher K values and lower DK values than low-grade HCCs.
Cao et al. [30]	2019	74 HCC	74	P	Predicting MVI and grading	TR = 5600 ms; TE = 63 ms; FOV = 380 × 289 mm^2^; matrix size = 100 × 76; ST = 6 mm; SG = 1 mm; FA = 90°, VS = 3.8 × 3.8 × 6 mm^3^	DKI	0, 200, 700, 1400, and 2100	DK, K	ROIs on the entire margin of the tumor on the slice where tumors showed their largest transverse diameter on the ADC maps, excluding areas of necrosis and hemorrhage by referring to T2w and T1w images; manual segmentation.	High-grade HCCs have higher mean K values and lower D values than low-grade HCCs. K showed better diagnostic performances for HCC grading and MVI prediction.
Jia et al. [45]	2019	151 patients with 182 hepatic nodules (114 HCCs, 33 FNHs, 29 hemangiomas, 6 HCAs)	114	R	Diagnosis	TR = 4500 ms, TE = 66 ms; FOV = 380 × 380 mm; matrix = 128 × 128; FA = 90; BW = 1954; ST = 6 mm, VS = NR	DKI	0, 200, 500, 800, 1500, and 2000	K, DK	ROIs on the level of maximum lesion transactional diameter, avoiding hemorrhage, necrosis, and cystic changes; manual segmentation.	HCC showed higher K and lower MD than benign nodules.
Yuan et al. [46]	2019	107 HCC	107	R	Response to treatment and prediction of recurrence	TR = 3300 ms; TE = 88 ms; FOV = 380 × 420 mm, FA = 90°, ST = 5 mm; SG = 1.5 mm; slices = 26; NEX = 3, VS = NR	DKI	0, 800, 1500, 2000 mm^2^/s	DK, K	ROIs on the largest HCC diameter; manual segmentation.	DK and K were able to predict recurrence of early stage HCC single nodules.
Kim et al. [31]	2019	180 (86 metastases, 61 HCC, 12 hemangioma, 10 simple cysts, 4 IHCC, 7 others)	61	R	Diagnosis	TE = 50.2 ms; TR = 5000 ms; FOV = 400 mm; matrix = 90 × 92, echo train length = 27; BW = 2877; ST = 5 mm, VS = NR	SE, IVIM	0,10, 25, 50, 75, 100, 200, 500, and 800	DDC, α, D, D*, f	ROIs on three consecutive slices of DWI images, including the largest lesion area; NR.	The DDC showed best performances for differentiating HCC from benign lesions.
Luo et al. [47]	2019	54 HCC	54	P	Response to treatment	TR = 4000 ms, TE = 88 ms, FOV = 380 × 380 mm, matrix = 128 × 128, ST = 6 mm, SG = 1.0 mm, VS = NR	DKI	300/500/1000	Dr, Da, DK, Ka, FAk, Kr, K	ROIs on the significant tumor enhanced area, avoiding iodized oil deposition and perivascular tissue; NR.	Dr, Da, and DK increased, and Ka and FAk decreased after TACE. No significant changes in FA, Kr, and K.
Server et al. [48]	2019	15 HCC	15	R	Response to treatment	TR = 2400 ms; TE = 82 ms; FOV between 240 and 380 mm, matrix of 115 × 192, EPI factor = 115; ST = 5 mm; SG = 1 mm, VS = NR	IVIM	0, 50, 100, 150, 200, 300, 400, 500, 600, 700, 800, 900, 1000, 1100, 1200, and 1300	D, D*, f	ROIs on the lesion with at least two-thirds of the lesions covered to prevent interference from the vascular and biliary structures; NR.	D increased and f decreased after treatment. Any significant results were found on D*.
Shao et al. [49]	2019	40 (20 IHCC, 20 HCC)	20	R	Diagnosis	TR = 9231 ms; TE = 56 ms FOV 38 × 30 cm; matrix size = 128 × 128; BW = 250 kHz; FA = 90; NEX acceleration factor 4, ST = 5 mm; SG = 1 mm; VS = NR	IVIM	0, 30, 50, 100, 150, 200, 300, 500, 800, 1000, and 1500	D, D*, f	ROIs on axial DWI images to encompass as much of the tumor as possible on the maximum tumor cross-section; manual segmentation.	D significantly higher and f lower in IMCC than in HCC. D* not significant.
Sokmen et al. [50]	2019	29 HCC	42	R	Grading	FOV between 240 and 380 mm, matrix = 115 × 192, ST = 5 mm; SG = 1 mm; vs. = NR	IVIM	0, 50, 100, 150, 200, 300, 400, 500, 600, 700, 800, 900, 1000, 1100, 1200, and 1300	D, D*, f	ROIs on DWI and IVIM images, covering at least two-thirds of the diameter of lesions to avoid blood vessels; NR.	High-grade HCCs had significantly lower D and higher f than low-grade HCCs, respectively. D values and f-values were negatively and positively correlated with HCC grade, respectively. The best discriminative parameter was f-value.
Wei et al. [51]	2019	115 HCC	135	P	Prediction of MVI	TR = 9230 ms; TE = 84.7 ms; FOV = 40 × 30 cm^2^; matrix = 80 × 128; ST = 6 mm; SG = 2 mm, VS = NR	IVIM	0, 10, 20, 40, 80, 100, 150, 200, 400, 600, 800, 1000, and 1200	D, D*, f	ROIs outlining the tumor margin on DWI images; manual segmentation.	D was higher in MVI-negative than in MVI-positive patients and is superior to ADC for evaluating the MVI of HCC. D* and f were not significant.
Zhang et al. [52]	2019	157 HCC	157	R	Prediction of recurrence	TR = 6000 to 10,000 ms, TE = 56 ms, FOV = 38 × 30 cm, matrix = 128 × 128, BW = 250 kHz/pixel, ST = 5 mm, SG = 1 mm, VS = NR	IVIM	0, 30, 50, 100, 150, 200, 300, 500, 800, 1000, and 1500	D, D*, f	ROIs encompassing much of the lesion as possible on DWI images, using T2WI as a reference; manual segmentation.	D is a potential biomarker for the preoperative prediction of recurrence after hepatectomy in HCC.
Wu et al. [53]	2018	55 HCC	55	P	Response to treatment and survival prediction	TR = 4100 ms; TE = 70 ms; FOV = 285 × 214–308 × 380 mm; matrix = 128× 128, ST = 6 mm, SG = 1 mm, vs. = NR	IVIM	0, 10, 20, 30, 40, 50, 70, 100, 200, 300, 500, and 800	D, D*, f (histogram analysis)	ROIs on IVIM sequences; semi-automatic segmentation.	F mean, median, and 25th percentile were higher, while skewness and kurtosis of PF were lower in responders than in non-responders.
Budjan et al. [54]	2018	56 patients with 68 hepatic lesions (25 HCC, 4 hepatic adenoma, 18 cysts, 18 hepatic hemangioma)	25	R	Diagnosis	TE = 75 ms, TR = 7800 ms; FOV = 340 × 240 mm; matrix = 192 × 140; FA = 90; GRAPPA 2; BW = 1644 Hz/pixel; ST = 4 mm, vs. = NR	DKI	50, 400, 800, and 1000.	DK, K	ROIs in the lesions excluding vessels and bile ducts; NR.	DK was able to distinguish HCC from benign lesions. K was not useful for lesion differentiation.
Hectors et al. [55]	2018	15 HCC	21	P	Diagnosis	TR = one respiration; TE = 75; FOV = 360 × 270 mm; matrix = 128 × 96, FA = 90; NEX = 1; ST = 7 mm; slices = 20; acceleration factor = 2, VS = NR	IVIM	0, 15, 30, 45, 60, 75, 90, 105, 120, 135, 150, 175, 200, 400, 600, and 800	D, D*, f	ROIs on the highest tracer activity of tumor. For normal liver, ROIs on left lobe and right lobe; manual segmentation.	IVIM parameters did not show significant differences between liver parenchyma and HCC.
Li et al. [56]	2018	41 HCC	41	P	Prediction of MVI	TR = 1973 ms; TE = 57 ms, FOV = 375 × 302 × 176 mm, matrix = 132 × 114, ST = 5 mm, SG = 0.5 mm, slices = 32, NSA = 2, vs. = NR	IVIM	0, 10, 20, 40, 80, 200, 400, 600, and 1000	D, D*, f (histogram analysis)	ROIs to encompass as much of the entire lesion in each slice, including cystic necrotic regions; NR.	D histogram features can be useful for predicting MVI. The 5th percentile of D was most useful value to predict MVI of HCC. The histogram parameters of D* and f showed no statistically significant differences between HCCs with and without MVI.
Wei et al. [57]	2018	65 HCC	68	R	Diagnosis	TR = 3750 ms; TE = 61.4 ms; FOV = 38 × 28 cm^2^; matrix = 128 × 128, ST = 5.0 mm, SG = 1 mm, VS = NR	IVIM	0, 10, 20, 40, 80, 100, 150, 200, 400, 600, 800, 1000, and 1200	D, D*, f	ROIs on the solid parts of the tumor; manual segmentation.	D is useful in differentiating ICC and HCC, while D* and f showed no significant results.
Zhao et al. [58]	2018	51 HCC	51	R	Prediction of MVI	TR = 5714 ms; TE = 65.5 ms; FOV = 38 × 28 cm^2^; matrix = 96 × 130; FA = 90; ST = 6 mm, NEX = 1, VS = NR	IVIM	0, 10, 20, 30, 40, 50, 60, 70,80, 90, 100, 200, 300, 400, 500, and 1000	D, D*, f	ROIs on the maximum representative slice in the tumor avoiding necrosis, cystic, hemorrhage, fat, fiber, blood vessels, and bile ducts; manual segmentation.	D was significantly lower in HCCs with MVI than HCCs without MVI and is an independent predictor of MVI.
Zhu et al. [59]	2018	62 HCC	62	R	Grading	TR = 4286 ms; TE = 61.2 ms; FOV = 38 cm × 28.5 cm; matrix size = 128 × 128, ST = 7 mm, SG = 1 mm, NEX = 6, 4, 2, 2, 2, 1, 1, 2, 4, 6, 6, 8, VS = NR	IVIM	10, 20, 40, 80, 100, 150, 200, 400, 600, 800, 1000, and 1200	D, D*, f	ROIs covering the largest solid part of the tumor; manual segmentation.	D was able to differentiate low-grade from high-grade HCC and was negatively correlated with histological grade. Any significant differences in D* values and f-values were found among the different HCC grades. D* was negatively correlated with HCC grade.
Wang et al. [60]	2018	84 HCC	92	P	Prediction of MVI	TR = 8000 ms; TE = 63 ms; FOV = 380 mm × 308 mm; matrix size = 128 × 128, ST = 5 mm, SG = 1 mm, VS = NR	DKI	0, 200, 500, 1000, 1500, and 2000	DK, K	ROI outlined around the tumor on ADC maps; manual segmentation.	Higher K values is a potential predictive biomarker for MVI of HCC.
Choi et al. [61]	2017	161 (91 HCCs, 27 IHCCs, 20 hemangiomas, 9 combined IHCC, 9 metastases, and 5 other tumors)	91	R	Diagnosis	TE = 60 ms; TR = 2100 ms; FOV = 340 × 256 mm; matrix size = 192 × 115, EPI factor = 115; BW = 1594 Hz; averages = 4; ST = 7 mm; SG = 1.4 mm; slices = 20, VS = NR	IVIM	0, 30, 60, 100, 150, 200, 400, 600, and 900	D, D*, f	ROIs on DWI images to covered the largest portion of the lesion; manual segmentation.	HCCs showed a significantly lower D than IHCC and a higher f than did IHCC and metastasis. No significant results concerning D*.
Luo et al. [62]	2017	27 (22 HCC, 5 FNH)	22	P	Diagnosis	TE = 60 ms; TR = 2100 ms; FOV = 340 × 256 mm; matrix = 192 × 115; EPI factor = 115; BW = 1594 Hz; averages = 4; ST = 7 mm; SG = 1.4 mm; slices = 20, VS = NR	IVIM	0, 30, 60, 100, 150, 200, 400, 600, and 900	D, D*, f	ROIs on the hepatic tumor in arterial phase T1w; manual segmentation.	D and D* were significantly lower in HCC in FNH, while f did not show any significant difference.
Shan et al. [63]	2017	106 HCC	109	R	Grading	TR = 9231, TE = 56; FOV = 38 × 30 cm^2^; matrix = 128 × 128; FA = 90, BW = 250, ST = 5 mm, SG = 1 mm; NEX = 1, VS = NR	IVIM	0, 30, 50, 100, 150, 200, 300, 500, 800, 1000, and 1500	D, D*, f	ROIs on b1000 DWI images to encompass as much lesion body; manual segmentation.	D and f were significantly different among well-, moderately, and poorly differentiated HCCs and were significantly correlated with histologic differentiation. No significant difference in D* value among the three groups.
Wu et al. [64]	2017	30 HCC	30	P	Response to treatment and survival prediction	TR = 54,100 ms; TE = 70 ms; FOV adapted patients’ body habitus, FOV = 285 × 214–308 × 380 mm; matrix = 128 × 128, ST = 6 mm, SG = 1 mm, vs. = NR	IVIM	0, 10, 20, 30, 40, 50, 70, 100, 200, 300, 500, 800	D, D*, f	ROIs on the whole lesion as large as possible to cover the viable and necrotic lesion part; NR.	D ratio 24–48 h after TACE was independent predictors for response to TACE for HCC and was associated with PFS.
Granata et al. [28]	2016	34 HCC	62	R	Grading	TR = 7500 ms; TE = 91 ms; FA = 90; matrix = 192 × 192, VS = NR	IVIM	0, 50, 100, 200, 400, 600, and 800.	D, D*, f	ROIs including hyper-intense voxels on b800 DWI images; manual segmentation.	D and f were statistically different in HCC groups with 1, 2, and 3 histological grade and were positively correlated with HCC grade.
Hectors et al. [65]	2016	25 HCC	37	P	Diagnosis	TR = one respiratory cycle, TE = 74–81 ms, FOV = 340–450 × 220–305 mm^2^, matrix = 160 × 80–132, reconstruction matrix = 320 × 100–256, EPI factor = 2, ST = 7 or 8 mm, VS = NR	IVIM	0, 15, 30, 45, 60, 75, 90, 105, 120,135, 150, 175, 200, 400, 600, and 800	D, D*, f	ROIs on HCC lesions and in the entire liver parenchyma; manual segmentation.	D, D*, and f were all significantly lower in HCC vs. liver parenchyma.
Murtz et al. [66]	2016	25 HCC	31	R	Response to treatment	FOV = 380×326 mm; matrix = 112 × 93; slice number = 28 BW = 1680.3 Hz; TE = 63 ms; TR = 1 respiratory cycle; imaging time per respiration = 1648 ms, ST = 7 mm; SG = 0.7 mm; VS = NR	IVIM	0, 50, 800	D, D*, f	ROIs on b800 DWI images as large as possible; manual segmentation.	D was increased after therapy, while f was decreased in responders. No significant changes were found in non-responders.
Kakite et al. [67]	2016	46 HCC	79	R	Prediction of necrosis	TR = 3000 ms; TE = 55–58, FOV = 80 × 128, FA = 90°, slice = 8; SG = 1.6 mm, acceleration factor 2, VS = NR	IVIM	0, 15, 30, 45, 60, 75, 90, 105,120, 135, 150, 175, 200, 400, 600, and 800	D, D*, f	ROIs on lesions and liver placed on T1W and copied to DWI images; NR.	D and f were higher in HCC than in liver. A significant correlation between D and f and tumor necrosis was found. D had the highest area under the curve for predicting complete tumor necrosis.
Klauss et al. [68]	2016	72 (29 FNH, 43 HCC)	43	P	Diagnosis	Slices = 14, ST = 5 mm, SG = 0.5 mm; acceleration factor = 2, vs. = NR	IVIM	0, 50, 100, 150, 200, 300, 400, 600, and 800	D, D*, f	ROIs on T1W, T2W and DWI; manual segmentation.	D were significantly lower in HCC compared to FNH and there was no significant difference for f and D*.
Shirota et al. [69]	2016	9 HCC	9	P	Response to treatment	TR = 1200 ms; TE = 63 ms; FOV = 400 × 454 mm, matrix = 110 × 110; FA = 90°; ST = 5 mm; BW = 921 Hz, VS = NR	IVIM	0, 50, 100, 150, 200, 400, and 800	D, D*, f	NR	Among IVIM metrics, only D of responders at baseline was significantly higher than that of the non-responders. Any significant results were found after treatment.
Goshima et al. [70]	2015	62 HCC	112	P	Response to treatment	TR = 2000 ms, TE = 67 ms; FOV = 380 × 304 mm; matrix = 256 × 256; acceleration factor = 2, ST = 6 mm, SG = 0 mm, VS = NR	DKI	0, 100, 500, 1000, 1500, and 2000	K	ROIs in entire HCCs and the surrounding liver parenchyma; NR.	K was able to assess post therapeutic response in HCC.
Qu et al. [71]	2015	53 patients with 85 liver masses (47 HCCs, 18 IHCC, 20 metastases)	47	P	Diagnosis	TR = variable, TE = 81.3 ms; FOV = 380 × 380 mm; matrix = 128 × 128, FA = 90, ST = 7 mm, VS = NR	IVIM	0, 50, 100, 250, 500, 750, 1000, and DWI 0, 700	D, D*, f	ROIs on the tumor, avoiding the hemorrhage and necrosis regions; NR	D was significantly higher in IHCCs than in HCCs and showed better performances than ADC. D* and f did not show significant differences between the two groups. No significant differences were found between HCCs and metastases.
Zhu et al. [72]	2015	55 (12 HV, 43 HCC)	23	P	Diagnosis	TR = 8571 ms; TE = 97.2–98.8 ms; FOV = 40 × 40 cm; FA = 90, ST = 8 mm; SG = 2 mm; number of excitations = 4; BW = 250 kHz, VS = NR	IVIM	10,20, 30, 50, 100, 200, 500, and 800	D, D*, f	ROIs at the S7 liver segment while avoiding the bile ducts and vessels (healthy group). For the patient group, ROIs on the b0 DWI images in restricted diffusion area, avoiding tumor vessels and necrosis; manual segmentation.	D was significantly lower in HCC than in hemangioma and healthy liver, but not different with respect to metastases. D* and f were not useful for HCC differentiation versus hemangioma, healthy liver, and metastases.
Park et al. [73]	2014	44 HCC	51	R	Response to treatment	TR = 1500 ms, FE = 70 ms, FOV = 284–300 × 284–301 mm; matrix = 192 × 108, FA = 90; ST = 5 mm; SG = 1 mm, vs. = NR	IVIM	0, 25, 50, 75, 100, 200, 500 and 800	D, D*, f	ROIs in any tumor area of lipiodol uptake or lipiodol defect; ROIs also on the adjacent non-tumorous hepatic parenchyma; manual segmentation.	D* values for HCC were significantly higher in lipiodol good uptake group than in poor uptake group. No significant differences were found on D and f.
Watanabe et al. [74]	2014	74 with 120 hepatic lesions (34 metastases, 32 HCC, 33 hemangiomas, and 21 liver cysts)	32	P	Diagnosis	TR = 1597 ms; TE = 55 ms; FOV = 380 × 304 mm, matrix = 112 × 90, FA = 90, BW = 59.3/7.32 Hz/pixel; EPI factor = 3; slices = 40; ST = 6 mm, SG = 0, VS = NR	IVIM	0, 10, 20, 30, 50, 80, 100, 200, 400, and 800	D, D*, f	ROIs encompassing the hepatic lesions on T1W, T2W, and contrast-enhanced MR images; manual segmentation.	D in hemangiomas and liver cysts were significantly greater than those of HCCs but were similar to those of metastases.
Woo et al. [75]	2014	40 HCC	42	R	Grading	TR = 5000ms; TE = 52 ms, FOV = 380 × 380 mm; matrix = 136 × 136; FA = 90, ST = 7 mm; SG = 0 mm, VS = NR	IVIM	0, 25, 50,75, 100, 200, 500, and 800 sec/mm^2^	D, D*, f	ROIs along the margin of the tumor on ADC maps, T2w, and T1 post contrast; manual segmentation.	D was higher in low-grade than in high-grade HCCs and was negatively correlated with histological grade.

## Supplementary Materials

The following are available online at https://www.mdpi.com/article/10.3390/jcm10122641/s1. Table S1: Characteristics of investigated non-gaussian DWI models. Abbreviations: IVIM = Intravoxel Incoherent Motion; DKI = Diffusion Kurtosis Imaging; SE = Stretched Exponential; NG = Non-gaussian; Table S2: Quality assessment using QUIPS tool; Table S3: Quality assessment using QUADAS-2 tool; Section S2: Key terms used in literature search.
