# Supplementary material for: The Role of Non-Gaussian Models of Diffusion Weighted MRI in Hepatocellular Carcinoma: A Systematic Review
Source: J Clin Med. 2021 Jun 15;10(12):2641. doi: 10.3390/jcm10122641 (PMC8232758; doi:10.3390/jcm10122641)
Supplement: Supplementary file 1 [file jcm-10-02641-s001.zip › jcm-1232607-supplementary.pdf]

The Role of Non-Gaussian Models of Diffusion Weighted MRI in Hepatocellular Carcinoma: A Systematic Review

S1. Non-Gaussian Diffusion Weighted MRI Models in HCC

Table S1. Characteristics of investigated non-gaussian DWI models. Abbreviations: IVIM = Intravoxel Incoherent Motion; DKI = Diffusion Kurtosis Imaging; SE = Stretched Exponential; NG = Non-gaussian.

NG Model	NG model Equation	No of NG parameters	NG parameters description
**IVIM**	$\frac{S(b)}{S_0} = (1-f) \cdot exp(-b \cdot D) + f \cdot exp(-b \cdot (D^* + D))$	3 (D, D*, f)	D = True Diffusion
			D* = Pseudo-diffusion Coefficient
			f = Perfusion Fraction
**DKI**	$\frac{S(b)}{S_0} = exp(-b \cdot D + \frac{b^2 \cdot {D_K}^2 \cdot K}{6})$	2 (K, DK)	K = Kurtosis Coefficient
			DK = Diffusion Coefficient Corrected for Kurtosis
**SE**	$\frac{S(b)}{S_0} = exp[-(b \cdot DDC)^{\alpha}]$	2 (DDC, α)	DDC = Distributed Diffusion Coefficient
			α = Heterogeneity Index

S2. Key terms used in literature search

- "HCC and non-gaussian diffusion MRI"
- "HCC and diffusion MRI"
- "HCC and DWI"
- "HCC and ADC"
- "HCC and IVIM"
- "HCC and intravoxel incoherent motion MR imaging"
- "HCC and biexponential diffusion model"
- "HCC and stretched exponential diffusion MRI"
- "HCC and DKI diffusion"
- "HCC and diffusion kurtosis imaging"
- "HCC detection and IVIM diffusion MRI"
- "HCC detection and biexponential diffusion MRI"
- "HCC detection and stretched exponential diffusion MRI"
- "HCC detection and diffusion kurtosis MRI"

- “HCC and grading and non-gaussian diffusion”
- “HCC and grading and IVIM”
- “HCC and grading and biexponential and diffusion”
- “HCC and grading and stretched exponential and diffusion”
- “HCC and grading and DKI”
- “HCC and prognosis and non-gaussian diffusion”
- “HCC and prognosis and IVIM”
- “HCC and prognosis and biexponential and diffusion”
- “HCC and prognosis and stretched exponential and diffusion”
- “HCC and treatment and DKI
- “HCC and treatment and IVIM”
- “HCC and treatment and biexponential and diffusion”
- “HCC and treatment and stretched exponential and diffusion”
- “HCC and treatment and DKI

S3. PRISMA Checklist

Section/topic	#	Checklist item	Reported on Page #
**TITLE**			
Title	1	Identify the report as a systematic review, meta-analysis, or both.	1
**ABSTRACT**			
Structured summary	2	Provide a structured summary including, as applicable: background; objectives; data sources; study eligibility criteria, participants, and interventions; study appraisal and synthesis methods; results; limitations; conclusions and implications of key findings; systematic review registration number.	1
**INTRODUCTION**			
Rationale	3	Describe the rationale for the review in the context of what is already known.	1-2

Objectives	4	Provide an explicit statement of questions being addressed with reference to participants, interventions, comparisons, outcomes, and study design (PICOS).	2
**METHODS**			
Protocol and registration	5	Indicate if a review protocol exists, if and where it can be accessed (e.g., Web address), and, if available, provide registration information including registration number.	NA
Eligibility criteria	6	Specify study characteristics (e.g., PICOS, length of follow-up) and report characteristics (e.g., years considered, language, publication status) used as criteria for eligibility, giving rationale.	2-3
Information sources	7	Describe all information sources (e.g., databases with dates of coverage, contact with study authors to identify additional studies) in the search and date last searched.	2-3
Search	8	Present full electronic search strategy for at least one database, including any limits used, such that it could be repeated.	2-3
Study selection	9	State the process for selecting studies (i.e., screening, eligibility, included in systematic review, and, if applicable, included in the meta-analysis).	2-3
Data collection process	10	Describe method of data extraction from reports (e.g., piloted forms, independently, in duplicate) and any processes for obtaining and confirming data from investigators.	3
Data items	11	List and define all variables for which data were sought (e.g., PICOS, funding sources) and any assumptions and simplifications made.	3
Risk of bias in individual studies	12	Describe methods used for assessing risk of bias of individual studies (including specification of whether this was done at the study or outcome level), and how this information is to be used in any data synthesis.	3
Summary measures	13	State the principal summary measures (e.g., risk ratio, difference in means).	NA
Synthesis of results	14	Describe the methods of handling data and combining results of studies, if done, including measures of consistency (e.g., I^2) for each meta-analysis.	NA
Risk of bias across studies	15	Specify any assessment of risk of bias that may affect the cumulative evidence (e.g., publication bias, selective reporting within studies).	NA
Additional analyses	16	Describe methods of additional analyses (e.g., sensitivity or subgroup analyses, meta-regression), if done, indicating which were pre-specified.	NA
**RESULTS**			
Study selection	17	Give numbers of studies screened, assessed for eligibility, and included in the review, with reasons for exclusions at each stage, ideally with a flow diagram.	3

Study characteristics	18	For each study, present characteristics for which data were extracted (e.g., study size, PICOS, follow-up period) and provide the citations.	4-18
Risk of bias within studies	19	Present data on risk of bias of each study and, if available, any outcome level assessment (see item 12).	18
Results of individual studies	20	For all outcomes considered (benefits or harms), present, for each study: (a) simple summary data for each intervention group (b) effect estimates and confidence intervals, ideally with a forest plot.	NA
Synthesis of results	21	Present results of each meta-analysis done, including confidence intervals and measures of consistency.	NA
Risk of bias across studies	22	Present results of any assessment of risk of bias across studies (see Item 15).	NA
Additional analysis	23	Give results of additional analyses, if done (e.g., sensitivity or subgroup analyses, meta-regression [see Item 16]).	NA
**DISCUSSION**			
Summary of evidence	24	Summarize the main findings including the strength of evidence for each main outcome; consider their relevance to key groups (e.g., healthcare providers, users, and policy makers).	20-22
Limitations	25	Discuss limitations at study and outcome level (e.g., risk of bias), and at review-level (e.g., incomplete retrieval of identified research, reporting bias).	21-22
Conclusions	26	Provide a general interpretation of the results in the context of other evidence, and implications for future research.	22
**FUNDING**			
Funding	27	Describe sources of funding for the systematic review and other support (e.g., supply of data); role of funders for the systematic review.	22

S4. Quality Assessment

Study	Year	Study participation	Study Attrition	Prognostic Factor Measurement	Outcome Measurement	Study Confounding	Statistical Analysis and Reporting
Jia et al	2020	LOW	LOW	LOW	LOW	LOW	LOW
Shi et al	2020	LOW	LOW	LOW	LOW	LOW	MODERATE
Hectors et al	2020	LOW	MODERAT E	LOW	LOW	LOW	MODERATE
Cao et al	2019	LOW	LOW	LOW	LOW	LOW	LOW
Yuan et al	2019	MODERATE	LOW	LOW	LOW	LOW	LOW
Luo et al	2019	MODERATE	LOW	LOW	LOW	LOW	MODERATE
Server et al	2019	MODERATE	LOW	LOW	LOW	LOW	MODERATE
Wei et al	2019	LOW	LOW	LOW	LOW	LOW	MODERATE
Zhang et al	2019	MODERATE	LOW	LOW	LOW	LOW	LOW
Wu et al	2019	LOW	LOW	LOW	LOW	LOW	MODERATE
Li et al	2018	LOW	LOW	LOW	LOW	LOW	LOW
Zhao et al	2018	MODERATE	LOW	LOW	LOW	LOW	LOW

Wang et al.	2018	LOW	LOW	LOW	LOW	LOW	LOW
Wu et al	2017	LOW	LOW	LOW	LOW	MODERATE	MODERATE
Murtz et al	2016	MODERATE	LOW	LOW	LOW	LOW	MODERATE
Kakite et al	2016	MODERATE	LOW	LOW	LOW	LOW	LOW
Shirota et al	2016	LOW	LOW	MODERATE	LOW	LOW	MODERATE
Goshima et al	2015	LOW	LOW	LOW	LOW	LOW	LOW
Park et al	2014	MODERATE	LOW	LOW	LOW	MODERATE	MODERATE

Table S2. Quality assessment using QUIPS tool

		Risk of bias				Applicability		
**STUDY**	**YEAR**	**PATIENT SELECTION**	**INDEX TEST**	**REFERENCE STANDARD**	**FLOW AND TIMING**	**PATIENT SELECTION**	**INDEX TEST**	**REFERENCE STANDARD**
Noda et al	2020	LOW	LOW	LOW	LOW	LOW	LOW	LOW
Peng et al	2020	UNCLEAR	LOW	LOW	LOW	LOW	LOW	LOW
Shan et al	2020	UNCLEAR	LOW	LOW	LOW	LOW	LOW	LOW
Whang et al	2020	LOW	LOW	UNCLEAR	LOW	LOW	LOW	LOW
Wu et al	2020	LOW	LOW	LOW	LOW	LOW	LOW	LOW
Wu B et al	2020	LOW	UNCLEAR	LOW	LOW	LOW	LOW	LOW
Jia et al	2019	UNCLEAR	LOW	UNCLEAR	LOW	LOW	LOW	LOW
Kim et al	2019	UNCLEAR	LOW	LOW	LOW	LOW	LOW	LOW
Shao et al	2019	UNCLEAR	LOW	LOW	LOW	LOW	LOW	LOW
Sokem et al	2019	UNCLEAR	UNCLEAR	LOW	LOW	LOW	LOW	LOW
Bujdan et al	2018	UNCLEAR	UNCLEAR	LOW	LOW	LOW	LOW	LOW
Hectors et al	2018	UNCLEAR	LOW	LOW	LOW	LOW	LOW	LOW
Wei et al	2018	UNCLEAR	LOW	LOW	LOW	LOW	LOW	LOW
Zhu et al	2018	UNCLEAR	LOW	LOW	LOW	LOW	LOW	LOW
Choi et al	2017	UNCLEAR	UNCLEAR	LOW	LOW	LOW	LOW	LOW
Luo et al	2017	UNCLEAR	LOW	LOW	LOW	LOW	LOW	LOW
Shan et al	2017	UNCLEAR	UNCLEAR	LOW	LOW	LOW	LOW	LOW
Granata et al	2016	UNCLEAR	LOW	LOW	LOW	LOW	LOW	LOW
Hectors et al	2016	LOW	LOW	LOW	LOW	LOW	LOW	LOW
Klauss et al	2016	LOW	UNCLEAR	LOW	LOW	LOW	LOW	LOW
Zhu et al	2015	LOW	UNCLEAR	LOW	LOW	LOW	LOW	LOW
Qu et al	2015	UNCLEAR	UNCLEAR	LOW	LOW	LOW	LOW	LOW
Watanabe et al	2014	LOW	LOW	LOW	LOW	LOW	LOW	LOW
Woo et al	2014	UNCLEAR	LOW	LOW	LOW	LOW	LOW	LOW

Table S3. Quality assessment using QUADAS-2 tool
